# The impact of electronic health records on the ordering of medical tests

**DOI:** 10.1186/s13584-025-00679-3

**Published:** 2025-03-31

**Authors:** Ofir Ben-Assuli, Doron Sagi, Sofia Amador Nelke, Moshe Leshno, Amitai Ziv, Avinoah Ironi

**Affiliations:** 1 Faculty of Business Management, Ono Academic College, 104 Zahal Street, 55000 Kiryat Ono, Israel; 2 MSR, Sheba Medical Center, Tel-HaShomer, Israel; 3 Ono Academic College, Kiryat Ono, Israel; 4 Tel-Aviv University, Tel-Aviv, Israel; 5 Sheba Medical Center, Tel-HaShomer, Israel

**Keywords:** Electronic health records, Medical test, Medical information

## Abstract

**Background:**

Healthcare facilities often encounter patients with incomplete records from previous visits, leading to duplicated tests. Recent Electronic Health Records (EHR) investments aim to address this issue. This study examines how viewing patient information via OFEK EHR affects the frequency of tests ordered by the physician. The OFEK system, developed in Clalit Health Services, is an advanced online medical records system used in hospitals. It was expanded to all hospitals and HMOs starting in 2013, allowing medical information to be shared and accessed in the Israeli healthcare system.

**Methods:**

The study was conducted at the Israel Center for Medical Simulation (MSR), with 26 physicians engaged in encounters with simulated patients (SP). The SPs provided relevant clinical histories and signs for two abdominal pain cases. The physicians ordered diagnostic tests, and after receiving the tests’ results they set a final diagnosis and could order additional tests. They had randomized access to the OFEK system to vary test-ordering patterns. In both scenarios, we examined three key variables to see if access to the OFEK system influenced the decision to order diagnostic tests (“QTestsBefore” – the number of tests ordered by the physician after the patient visit; “QTestsAfters” – the number of tests ordered by the physician after receiving the results of the first round; “QSumTests” – The total number of tests).

**Results:**

In the study group with access to the OFEK EHR, an average of 5.5 tests were ordered, compared to 6.85 in the control group (*p*-value = 0.01). Ordinary Least Squares regressions confirmed that the overall number of tests, particularly the second round ordered after receiving initial results, was significantly lower with OFEK. Additionally, years of clinical practice also correlated with fewer ordered tests.

**Conclusions:**

The findings show that the OFEK EHR system reduces the number of medical examinations by allowing physicians to access medical histories and past tests, which supports efficient decision-making. This leads to fewer ordered medical tests and, thus, reduces the time procedures patients spend in EDs or hospitals. Efficient decision-making and fewer redundant medical tests can improve patient flow, free up resources, and reduce overcrowding in emergency departments.

## Introduction and background

The healthcare sector has recently invested in many clinical technologies (Atasoy et al. 2018; Ben-Assuli et al. 2015), including Electronic Health Records (EHRs), to improve patient care and decision-making. EHR systems enhance healthcare applications by increasing quality and security and offer significant economic benefits (Goetz et al. 2012; Haleem et al. 2022; Yaraghi et al. 2014). EHRs are crucial in improving medical procedures (Ayabakan et al. 2017; Bardhan et al. 2020), supporting timely and accurate decision-making, and addressing complex healthcare challenges (Hripcsak et al. 2013; Miriovsky et al. 2012; Reges et al. 2020). By providing complete, real-time patient information, EHRs facilitate higher quality, safer, and more cost-effective treatments, ultimately streamlining healthcare delivery (Braga et al. 2015). The widespread use of EHRs saves approximately $7.9 billion annually by reducing redundant diagnostic tests specifically (Hillestad et al. 2005).

Recognizing these benefits, Clalit Health Services, Israel’s largest Health Maintenance Organization (HMO), introduced the OFEK EHR system in 2005 to enhance care and reduce costs (Nirel et al. 2011). OFEK facilitates comprehensive data-sharing between hospitals and HMO systems, including diagnostic test results, imaging, and treatment records (Oderkirk 2017). Expanded nationally in 2013 under the Ministry of Health’s directive, OFEK became a unified system for securely sharing medical data, transforming patient care across Israel (Gefen et al. 2019). A previous study demonstrated that the use of OFEK significantly improved diagnostic accuracy (Ben-Assuli et al. 2015), highlighting its potential impact on clinical decision-making.

To assess the practical impact of EHR integration on clinical decision-making, simulations were conducted at the Israel Center for Medical Simulation (MSR). MSR is a national training center that uses simulation to enhance healthcare professionals’ clinical skills. The training was conducted by simulated patients (SPs), and trained actors who authentically replicated emergency scenarios, including high-stress levels and typical emergency department (ED) patient responses, ensuring consistency and realism in the study environment (Ziv et al. 2005; Ziv et al. 2006).

A key issue in modern healthcare is the redundancy of medical tests due to not effective information-sharing (Ayabakan et al. 2017). A study found that only 3–20% of physicians communicate with patients’ primary care providers, while 33–63% of discharge summaries lack essential details like diagnostic results (Kripalani et al. 2007). These gaps contribute to delays, patient dissatisfaction, safety risks, and increasing the likelihood of repeated procedures. The problem is worsened by the fragmented healthcare IT infrastructure, where disparate systems fail to communicate effectively across or even within organizations (Aceto et al. 2018; Wager et al. 2017). This all leads to the understanding that decision-making is hindered without seamless access to medical histories, and thus, healthcare efficiency declines (Ben-Assuli et al. 2015).

This study investigated the impact of using the OFEK system on the number of ordered diagnostic tests. We assumed that a lack of information regarding patients’ medical history would increase the number of tests compared to cases where this information is accessible. We also compared physicians according to their experience.

## Methodology

### The experiment and medical scenarios

The study was conducted at the MSR Simulation Center, designed to replicate an ED environment. Twenty-six physicians, voluntarily participated during their work hours. Each physician took part in two distinct Simulated Patient (SP) cases, cumulating in 52 simulations overall. The scenarios reflected common clinical situations identified by the National Center for Health Statistics (NCHS),^1^ and their scripts were developed by senior ED physicians and MSR simulation and assessment experts.

The SPs were professional actors meticulously trained to replicate emergency cases. They provided uniform presentations, including clinical histories, emotional cues, and observable physical signs, ensuring consistency across all simulations. To enhance realism, SPs exhibited high-stress levels typical of ED patients and provided physiological indices aligned with their portrayed illnesses.

In Scenario A, the SP presented with severe, prolonged abdominal pain. Information stored in the OFEK EHR system supported a diagnosis of irritable bowel syndrome (IBS), potentially avoiding unnecessary tests and hospitalization. In Scenario B, the SP reported mild hip pain radiating to the left leg, raising suspicion of spinal or renal issues. Access to OFEK allowed physicians to review prior CT scans demonstrating dilation of the abdominal aorta, leading to immediate actions for accurate diagnosis and treatment for abdominal aortic aneurysm. Both scenarios included a clear clinical outcome and differential diagnosis as benchmarks.

**Fig. 1:**
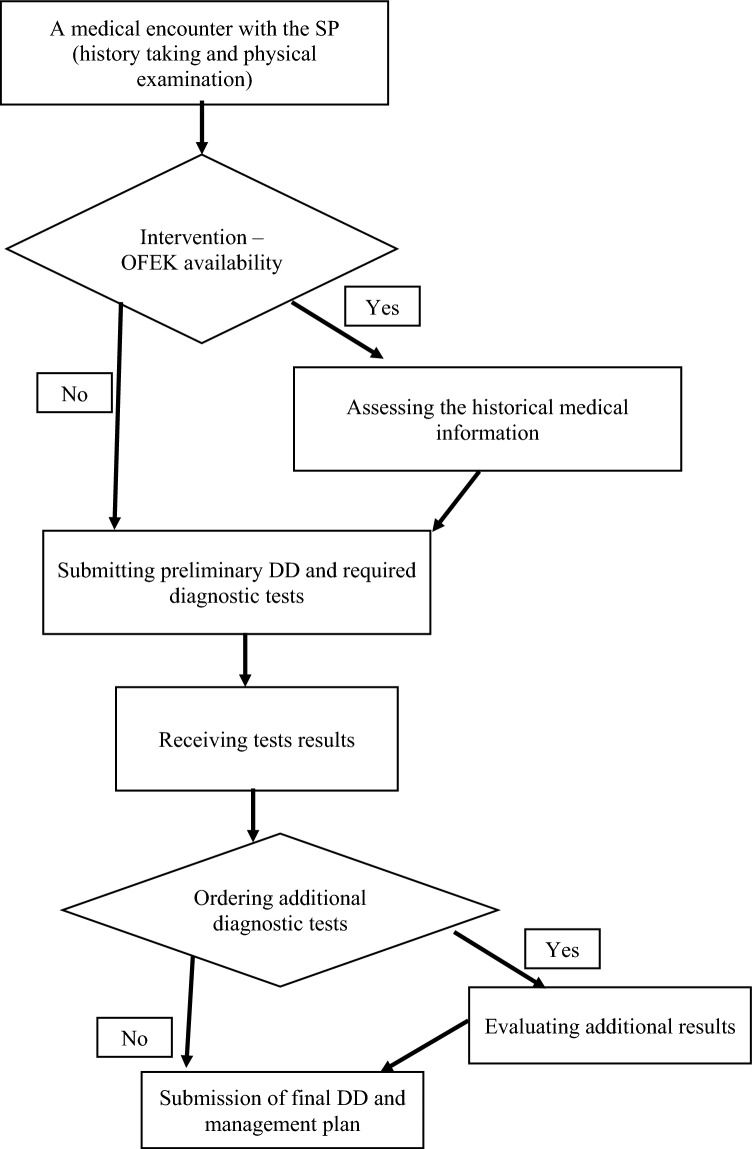
The flowchart of the possible paths (decision) during the experiment

All scenarios had a standard course of events: (1) Physicians began by a medical encounter with the SP, taking their medical history, and conducting physical examination without time limits. (2) After leaving the room, they stated their preliminary differential diagnosis and ordered diagnostic tests (a second round of tests was optional after receiving the results of the first round). (3) In the last phase, the physicians received the test results (prepared in advance) and stated their final differential diagnosis, decision to admit or discharge, and management plan. Since the second stage, physicians in the study group were encouraged to use OFEK until their final decision (See Fig. 1). Time to handle the case was measured from the start of the encounter until the submission of the final DD and the management plan. For each scenario, physicians were randomized to access or not access the OFEK system. The OFEK system in this study was the commercial version of the system, with mock medical files for the simulated patients. The use of OFEK was obligatory for the study group, but we did not control or oversee their search, so the information extracted from OFEK and the underlying decision may vary.^2^ This experimental setup allowed for a controlled evaluation of how access to comprehensive patient information via the OFEK influences diagnostic decision-making and test ordering behavior.

### Dependent variables

Three medical test ordering variables were examined.

“*QTestsBefore*” – Measured the number of medical tests that the physician ordered after seeing the patient and receiving the first evaluation of the patient.

“ *QTestsAfters*” – Assessed whether a second round of tests was ordered after receiving the results of the first round.

“*QSumTests*” – The total number of tests (*QTestsBefore* + *QTestsAfter*).

### Independent variables

OFEK: This variable was coded as dichotomous: 1 for full access to the EHR and 0 for no access. The system used was the commercial version of OFEK. All physicians had prior experience using the system. Background medical information was intentionally included in the EHR designed by MSR experts. Physicians accessed the information by entering the patient’s simulated ID number. In this experiment, not all of the patient’s history was included in OFEK; it was restricted to general health information and history relevant to the simulation.

Time: This factor significantly influences decision-making, especially in the Eds (Ahituv et al. 1998; Walzl et al. 2022). It was measured in minutes to represent the time each physician needed to handle a case, including the time to assess the patient's condition at each medical stage.

Seniority: Previous research indicates that professionals at different levels may have distinct decision-making processes (Cohen et al. 2013; Dew et al. 2009; Salas et al. 2010). This study classified physicians based on their seniority (1 = senior physicians, 0 = residents).

Specialty: Physicians were classified according to their specialty, as studies have indicated that physicians from different specialties may use different information components. Internists were coded as 1, and emergency physicians as 0.

Years of practice: The number of years a physician has been practicing correlates with experience and clinical expertise. Physicians with more years of practice have likely encountered a wider range of medical cases, which could influence their decision-making processes, including their reliance on diagnostic tests.

Gender: 1 for male and 0 for female.

### Statistical analyses

The statistical analyses were performed using Python.

All data are presented as a mean ± SD for the continuous variables and as a percentage for categorical variables. Categorical variables were compared between groups using the chi-square. Continuous variables were compared using a t-test. The relevant statistical tests were performed per variable.

Ordinary Least Squares (OLS) regression analysis was employed to investigate the relationship between access to the OFEK EHR system and the number of diagnostic tests ordered. OLS is a statistical method designed to estimate relationships between dependent variables, such as QTestsBefore, QTestsAfter, and QSumTests, and independent variables, including EHR access, physician specialty, seniority, and years of experience. The method minimizes the sum of squared residuals to produce the best-fitting linear relationship, making it particularly suitable for analyzing continuous dependent variables while controlling for the effects of other factors. In this study, OLS was chosen to assess how various factors influence the number of medical tests ordered during the simulation, given that the dependent variables are continuous.

## Results

### Descriptive statistics

The study involved 26 physicians, 15 internists (58%), and 11 emergency physicians, as seen in Table 1. Among these, 7 (27%) were senior physicians, and the remaining 19 were residents. The sample had an almost equal distribution of male (54%) and female participants. Most participants were residents, which mirrors the typical staffing in hospital EDs, where residents usually outnumber senior physicians. The sample covered a wide range of professional backgrounds, enabling a meaningful analysis of how factors, such as specialty and seniority, influence clinical decision-making.

**Table 1:** Descriptive statistics of independent variables

Variables	Mean
OFEK EHR	26 cases (50%)
Specialty (% Internists)	15 physicians (58%)
Seniority (% Senior physicians)	7 physicians (27%)
Age	36.7 ± 3.7
Time	10.5 ± 3.8
Gender (% Male)	14 physicians (54%)
Years of practice	3.9 ± 3.2

Table 2 shows the descriptive statistics of the dependent variables. On average, physicians ordered 3.96 tests initially (QTestsBefore) with a standard deviation of 1.72, ranging from 1 to 8 tests, showing moderate variability in initial test ordering. For follow-up tests (QTestsAfter), the mean was 2.21, with most physicians ordering between 1 and 3 follow-up tests. The total number of tests ordered (QSumTests) averaged 6.17, with a standard deviation of 2.14 and a range of 2–11 tests, indicating variability in overall test-ordering behavior. These results provide a baseline for analyzing the impact of the OFEK EHR system.

**Table 2:** Descriptive statistics of dependent variables

	Mean
QTestsBefore	3.96 ± 1.72
QTestsAfter	2.21 ± 1.09
QSumTests	6.17 ± 2.14

Table 3 presents the comparative general statistics of the study group (with access to the OFEK EHR) and the control group (without access) across the variables. In terms of specialty, internists accounted for 62% of the study group compared to 54% in the control group. For seniority, senior physicians represented 19% of the study group versus 35% in the control group, while the rest were residents. All these differences were insignificant. Regarding average age, the study group physicians were slightly older (37.13 ± 4.3 years) than the control group (36.27 ± 3.27 years), although this difference was not statistically significant (*p* = 0.43).

**Table 3:** General statistics of study group versus control group

Variable	Study group with access to the EHR	Control group without access to the EHR	Test value	*P*-value	95%-CI (Confidence interval)
Specialty			${\chi }^{2}=0.315$	0.575	–
Internist Emergency physicians	16 (62%) 10 (38%)	14 (54%) 12 (46%)			
Seniority			${\chi }^{2}=1.564$	0.211	–
Senior physicians Residents	5 (19%) 21 (81%)	9 (35%) 17 (65%)			
Age, years ± SD	37.13 ± 4.3	36.27 ± 3.27	$t=-\hspace{0.17em}0.791$	0.43	[− 3.04–1.33]
Time to handle a case, in minutes ± SD	9.69 ± 3.3	11.38 ± 4.08	$t=1.652$	0.1	[− 0.37–3.75]
Gender			${\chi }^{2}=0.315$	0.389	–
Male Female	14 (58%) 10 (42%)	12 (46%) 14 (54%)			
Years of practice ± SD	3.46 ± 3.35	4.59 ± 3.03	$t=1.228$	0.23	[− 0.72–2.98]
QTestsBefore ± SD	3.58 ± 1.6	4.35 ± 1.77	$t=1.644$	0.1	[− 0.17–1.7]
QTestsAfter ± SD	1.92 ± 1.06	2.5 ± 1.07	$t=1.959$	0.56	[− 0.01–1.7]
QSumTests ± SD	5.5 ± 1.97	6.85 ± 2.13	$t=2.369$	0.01	[0.21–2.49]

The average time required to handle a case was shorter in the study group (9.69 ± 3.3 min) than in the control group (11.38 ± 4.08 min), showing a trend toward efficiency with EHR access (*p* = 0.1). Male participants were slightly more represented in the study group (58%) than in the control group (46%). All these differences were also insignificant.

Notably, the study group ordered fewer initial tests (QTestsBefore: 3.58 ± 1.6) and follow-up tests (QTestsAfter: 1.92 ± 1.06) than the control group (4.35 ± 1.77 and 2.5 ± 1.07, respectively). The total number of tests (QSumTests) was significantly lower in the study group (5.5 ± 1.97) compared to the control group (6.85 ± 2.13), *p* = 0.01. These findings suggest that access to the OFEK system reduces test ordering, highlighting its potential for improving diagnostic efficiency and resource utilization.

### Impact of using OFEK on the number of test orders

Tables 4, 5, 6 show the OLS results for the following dependent variables: QTestsBefore, QTestsAfter, and QTestsSum. In Table 4, the OFEK system did not significantly impact the number of medical tests ordered in the first stage. The negative coefficient suggests that access to OFEK slightly reduces the number of tests ordered after the initial evaluation. Still, this effect is not strong enough to be considered statistically significant (*p* = 0.31).

**Table 4:** OLS regression results for dependent variable QTestsBefore

Variable	Beta	*T*-test value	*p*-value	95%-CI (Confidence interval)
Const	7.7476	2.521	0.015	[1.555–13.94]
OFEK	− 0.5062	− 1.026	0.31	[− 1.5–0.49]
Specialty	0.6248	1.209	0.233	[− 0.416–1.666]
Seniority	0.7263	0.867	0.39	[− 0.961–2.414]
Age	− 0.0949	− 1.035	0.306	[− 0.28–0.09]
Time	0.0288	0.462	0.646	[− 0.097–0.154]
Gender	− 1.0539	− 2.011	0.05	[− 2.11–0.002]
Years of practice	− 0.0894	− 0.795	0.431	[− 0.316–0.137]

$${R}^{2}$$=0.254

**Table 5:** OLS regression results for dependent variable QTestsAfter

Variable	Beta	*T*-test value	*p*-value	95%-CI (Confidence Interval)
Const	0.6579	0.339	0.736	[− 3.249–4.565]
OFEK	− 0.8083	− 2.599	0.013*	[− 1.435 to − 0.181]
Specialty	− 0.3485	− 1.069	0.291	[− 1.005–0.308]
Seniority	0.5772	1.093	0.281	[− 0.487–1.642]
Age	0.0663	1.147	0.258	[− 0.05–0.183]
Time	− 0.0098	− 0.25	0.804	[− 0.089–0.069]
Gender	0.6505	1.968	0.055	[− 0.016–1.317]
Years of practice	− 0.1758	− 2.48	0.017*	[− 0.319 to − 0.033]

$${R}^{2}$$=0.267

**Table 6:** OLS regression results for dependent variable QSumTests

Variable	Beta	*T*-test value	*p*-value	95%-CI (Confidence Interval)
Const	8.4055	2.14	0.038	[0.491–16.32]
Ofek	− 1.3145	− 2.086	0.043*	[− 2.585 to − 0.044]
Specialty	0.2763	0.418	0.678	[− 1.054–1.607]
Seniority	1.3035	1.218	0.23	[− 0.853–3.46]
Age	− 0.0286	− 0.244	0.809	[− 0.265–0.208]
Time	0.0189	0.238	0.813	[− 0.141–0.179]
Gender	− 0.4034	− 0.603	0.55	[− 1.753–0.946]
Years of practice	− 0.2652	− 1.846	0.072^+^	[− 0.555–0.024]

$${R}^{2}$$=0.218

However, in Table 5, the OFEK system significantly negatively affects the number of tests ordered after the initial test results are received. It indicates (*p* = 0.013) that physicians with access to OFEK are statistically less likely (negative coefficient (− 0.8)) to order additional tests after seeing the results from the first round of tests. This suggests that when physicians can review a patient’s previous records and medical history through the OFEK system, they are more confident in making decisions based on available information, thus reducing unnecessary follow-up testing.

In addition, experienced physicians ordered fewer follow-up tests (*p* = 0.017), which indicated that they might be more confident in making decisions based on initial assessments or the available data.

As Table 6 shows, the OFEK system also significantly affects the total number of tests ordered (QSumTests). The negative coefficient (− 1.31) indicates that access to OFEK reduces the total number of tests ordered during the simulation. The *p*-value of 0.043 confirms that this effect is statistically significant.

## Discussion

Physicians often rely on diagnostic test results to make critical medical decisions (Bashkin et al. 2015; Land et al. 2019; McDowell et al. 2018). Early diagnosis and timely, accurate medical information significantly enhance the likelihood of effective treatment and rapid recovery (Castaneda et al. 2015). However, choosing the most appropriate test can be challenging, as diagnostic processes are rarely documented and not always research-based (di Ruffano et al. 2017). This issue is further complicated when patients fail to provide a comprehensive medical history or accurately describe their symptoms, assuming their health information is readily accessible across providers (Ben-Assuli and Leshno 2012). Miscommunication or poor recall of prior treatments and test results often leaves providers without the necessary information, leading to repeated diagnostic tests (Blease and Bell 2019).

The findings of this study reinforce the significant impact of EHR on medical decision-making efficiency by reducing the number of redundant diagnostic tests. The use of the OFEK system showed a significant decrease in tests ordered after the first round (QTestsAfter) and in the total number of tests ordered (QSumTests), which aligns with previous research suggesting that access to comprehensive patient information through EHRs can enhance diagnostic accuracy and reduce unnecessary procedures (Ben-Assuli et al. 2015; Hillestad et al. 2005). By consolidating medical histories in a single, easily accessible platform, EHRs ensure that physicians have complete patient information at the point of care, reducing reliance on patient recollection and minimizing redundant testing.

Additionally, clinical experience played a crucial role in test-ordering behavior. Physicians with greater clinical experience ordered fewer follow-up tests than their less experienced counterparts, underscoring the importance of clinical experience in diagnostic decision-making. However, EHRs have the potential to mitigate this experience gap, providing less experienced physicians with a critical decision-support tool (Vasanthakumar et al. 2024). By offering access to a detailed and consolidated medical history, EHRs enable less experienced physicians to make more confident and informed decisions (Graber et al. 2017). This is particularly crucial in high-pressure environments such as EDs, where time constraints often complicate decision-making. EHRs could serve as an equalizer by reducing the reliance on experience alone and promoting consistency in diagnostic practices across all physician levels (Weiskopf and Weng 2013).

Furthermore, the synergy between EHR access and clinical experience could further optimize resource utilization and improve patient care. Experienced physicians could leverage EHRs to refine their decision-making processes further, while junior physicians could use them to bridge the experience gap, ensuring high-quality patient care. A study comparing advanced practice clinicians (APCs) and primary care physicians (PCPs) found that APCs were associated with more imaging orders than PCPs, highlighting how experience and role influence test-ordering practices. This finding supports the idea that EHRs serve as a balancing tool, ensuring that all clinicians—regardless of seniority—have access to the same comprehensive patient information, leading to more standardized and efficient decision-making (Hughes et al. 2015).

### Implication of the study

One of the key implications of this study is the economic benefit of reducing unnecessary medical tests. The healthcare industry, particularly in EDs, often faces resource constraints, and redundant testing increases operational costs and burdens healthcare systems. The findings suggest that EHRs like the OFEK system can reduce these costs by avoiding duplicate or unnecessary tests, resulting in a more efficient allocation of hospital resources and staff. This aligns with prior studies indicating that the widespread use of EHRs could save billions annually by reducing redundant diagnostics (Hillestad et al. 2005).

Moreover, time-saving is a crucial benefit observed in this study. Physicians with access to OFEK could make quicker decisions, resulting in faster patient throughput and less time spent in EDs. This reduction in the length of stay (LOS) directly addresses a critical issue in emergency care—overcrowding. By shortening the time needed to gather and analyze patient information, EHRs can help alleviate ED congestion, which has been linked to improved patient outcomes (Oh et al. 2018).

Another important implication is the potential reduction in hospital-acquired infections (HAIs) (Bulmash et al. 2020), particularly for immunocompromised patients who frequently return to the hospital. These patients are at greater risk of contracting HAIs during repeated or prolonged hospital stays. By reducing the number of hospital visits and the time patients spend in healthcare settings through faster diagnoses and more efficient care, EHRs like OFEK can indirectly lower the risk of HAIs, supporting broader infection control efforts in hospitals (Blot et al. 2022).

Additionally, this study’s demonstrated simulation’s significant methodological advantage, providing a more in-depth analysis than other evaluation methods (Cook et al. 2011). Simulations allow for controlled, replicable scenarios where specific variables can be isolated and studied systematically (Barry Issenberg et al. 2005; Cheng et al. 2016). Simulation provides a realistic and controlled environment to test the implementation and the impact of new healthcare technologies. Unlike retrospective analyses or observational studies, simulations enable real-time assessment of physician decision-making processes in dynamic and high-pressure environments, such as those mimicking ED scenarios (Ilgen et al. 2013).

Finally, as we move forward into using more and more Internet of Things (IoT) in the medical field (Lederman et al. 2021). The integration of IoT devices with EHR can enhance patient care and clinical decision-making (Nakhla and Nouira 2024). By enabling continuous monitoring through smart biomedical devices, healthcare providers can access real-time data on patients’ vital signs and health metrics, leading to more personalized and timely interventions (Barbieri et al. 2023; Ghosh et al. 2023). This IoT data flow into EHRs not only improves the accuracy of medical records but also facilitates proactive healthcare delivery, potentially reducing hospital admissions and improving patient outcomes (Ranjan and Ch 2024).

## Conclusion

This study showcases the effectiveness of Electronic Health Records, specifically the OFEK system, in decreasing unnecessary medical tests, thus improving both economic efficiency and patient care. The results indicate that having access to detailed patient histories decreases the amount of diagnostic tests that the patient has to take. This, in turn, may decrease the hospital time for the patients, facilitate faster, more informed decision-making and diagnoses, and potentially lower rates of hospital-acquired infections. These advantages underscore the necessity for ongoing investment in and growth of EHR systems to improve healthcare delivery.

The findings are particularly relevant to EDs, where resource constraints and time pressures are critical concerns. The OFEK system’s capacity to reduce redundant tests and to speed up decision-making indicates that broader implementation of EHRs could assist in addressing challenges such as ED overcrowding, patient dissatisfaction, and healthcare costs.

Overall, this study underscores the importance of EHRs in modern healthcare systems. While the findings are promising, future research should explore the system's impact on different clinical scenarios and assess the effectiveness of other EHR platforms. Additionally, further studies could investigate how factors such as physician training and the usability of the EHR interface affect the system's effectiveness in reducing redundant medical tests.

## Future research and research limitations

Nowadays, hospitals and healthcare providers worldwide use EHRs. Although EHRs clearly have significant benefits, it is difficult to determine their effectiveness. This study's findings suggest that using the information provided in EHRs reduces the number of tests ordered and contributes to the quality of physicians' decision-making. However, this study has several limitations. Firstly, it occurred in a simulated environment, which was not identical to a real environment with multiple distractions. The scenarios were based on real cases but performed by actors. Secondly, the study only dealt with cases of abdominal pain, which may limit its generalizability. Additionally, as a proof-of-concept study, its findings are constrained by the small sample size of 26 physicians, reducing the statistical power and the ability to draw broader conclusions. A larger, more diverse sample would help validate the results and improve their applicability to real-world settings. Physicians in the study group had the opportunity to use the OFEK. In real-life time constraints, a lack of knowledge and experience navigating the OFEK may prevent physicians from using this EHR. Training and understanding of the OFEK contribution to the ED and to patient care are required as part of the training program of ED physicians.

Future research could consider other medical problems to test whether EHRs contribute in the same way in different medical scenarios. Additionally, other EHR systems should be included in further research, as they might differ in their user interface and functionalities from the OFEK system, potentially impacting the user experience and leading to different results. Furthermore, future research should examine different EHR systems in terms of actual system components, such as user-friendliness, functionalities, and user interface, to assess their impact on test ordering and physician decision-making. Addressing these limitations in future studies will enhance the reliability and generalizability of findings, providing a more substantial evidence base for implementing EHR systems in diverse healthcare contexts.

## Data Availability

The data supporting this study's findings are available for viewing on a video conference by request from the corresponding author.

## List of Abbreviations

*APC*: Advanced practice clinician
*ED*: Emergency department
*EHR*: Electronic health records
*HAI*: *H*ospital acquired infections
*HMO*: Health Maintenance Organization
*IBS*: Irritable bowel syndrome
*LOS*: Length of stay
*MSR*: Israel Center for Medical Simulation
*OLS*: Ordinary least squares
*NCHS*: National Center for Health Statistics
*PCP*: Primary care physician
*SP*: Simulated patient

## Footnotes

1 https://www.cdc.gov/nchs/index.html

2 The study environment was designed to resemble routine ED conditions. Physicians were excused from their shifts for the simulation and treated the SPs as part of their daily workflow. Although there were no imposed time limits, they could return to their regular duties and revisit the simulation if necessary. The simulated environment was equipped with the national commercial version of the OFEK EHR system to compare decision-making between groups, which contained mock medical files tailored to the scenarios. Access to OFEK did not guarantee the availability of useful information, mirroring real-world variability in EHR data relevance. For a detailed description of the simulation see Ben-Assuli et al. 2015.
